# Constrained Antenna Selection and Beam Pointing Control for Directional Flying Ad Hoc Networks

**DOI:** 10.3390/s26051635

**Published:** 2026-03-05

**Authors:** Xiangrui Fan, Shuo Zhang, Wenlong Cai, Shaoshi Yang

**Affiliations:** 1 Department of Aerospace Science and Technology, Space Engineering University, Beijing 101400, China; 2 Beijing Aerospace Automatic Control Institute, Beijing 100854, China; 3 Joint Laboratory of Aerospace Control and Communication Technology, Beijing 100876, China; 4 School of Information and Communication Engineering, Beijing University of Posts and Telecommunications, Beijing 100876, China

**Keywords:** FANETs, directional networking, antenna selection, beam pointing control

## Abstract

With the increasing complexity of the space electromagnetic environment, traditional omnidirectional antenna-aided communication and networking techniques can no longer meet the collaboration requirements of aircraft clusters. To achieve goals such as anti-jamming, anti-interception, and enhanced spatial multiplexing, an increasing number of aircraft are being equipped with high-gain directional antennas. However, modeling of directional antenna-constrained Flying Ad Hoc Networks (FANETs) is far more complex than modeling of omnidirectional antenna-aided networks. The former task is highly dependent on the real-time flight state and the spatial topology of the network. In response to the communication challenges posed by directional networking of highly-dynamic aircraft clusters, this study proposes an antenna selection and beam pointing control algorithm, which is deeply integrated with the aircraft’s Guidance, Navigation, and Control (GNC) system. By introducing parameters that characterize dynamic flight state, such as position and attitude information, and combining them with high-precision multi-coordinate system transformations and spatial geometric analysis methods, the proposed algorithm enables the real-time optimization of antenna selection and beam pointing under the relative motion trends of aircraft. It effectively maintains high-quality connections between flying nodes. Digital simulation and physical experiment results demonstrate that the proposed method can accurately calculate the appropriate antenna selection and determine precise beam pointing directions based on the position data of flying nodes. This provides an important reference for the design of optimized communication strategies used in directional networking of highly-dynamic aircraft clusters.

## 1. Introduction

Unmanned Aerial Vehicle (UAV) clusters, as an emerging technological paradigm, demonstrate significant advantages over single aircraft when executing complex missions. These advantages include higher survivability, stronger mission execution efficiency, and dynamic self-organization and self-recovery capabilities. To fully leverage these benefits, the construction of an efficient and reliable inter-UAV communication network is of paramount importance, which has given rise to the research field of FANETs. As an extension of Mobile Ad Hoc Networks (MANETs) into three-dimensional space, FANETs are characterized by their highly-dynamic property, lack of centralization, and rapid topology changes, posing unprecedented challenges to communication technologies.

In traditional FANETs research, omnidirectional antenna models have been widely adopted. However, these models have revealed their inherent limitations in the increasingly complex electromagnetic environment. The dispersed energy radiation of omnidirectional communication results in limited transmission ranges between nodes, making it difficult to support long-range collaborative missions. More critically, omnidirectional signals render the network highly susceptible to external interference, and information is easily intercepted, severely compromising the secrecy and security of missions [1]. To address these issues, researchers have recently turned to the more advantageous directional antenna technology. Directional antennas, by focusing energy radiation in specific directions, significantly enhance the signal’s resistance to interference and interception, improving the link’s secrecy and security [2]. Moreover, through efficient spatial multiplexing, directional antennas effectively reduce mutual interference between nodes, thereby substantially increasing network throughput. However, the application of directional antenna technology also brings new technical challenges [3]. The FANETs model under directional connectivity constraints is essentially more complex than the omnidirectional model [4], with this complexity stemming from the model’s high dependence on the real-time flight status and spatial topology of network nodes. It presents significant challenges in the design of networking communication protocols, such as neighbor discovery, multiple access protocols, and routing algorithms [5].

### 1.1. Related Work

To tackle these challenges, researchers are actively engaged in the exploration of novel communication protocols and algorithms for FANETs under directional connectivity constraints, aiming to adapt to the connectivity limitations and dynamic topology changes imposed by directional antennas.

For example, in addressing the neighbor discovery problem based on directional antennas, Hong et al. [6] propose a Chinese Remainder Theorem (CRT)-based Multi-Antenna Neighbor Discovery (MAND) algorithm to achieve faster beam alignment within bounded latency. Bai et al. [7] introduce a cognitive framework to minimize the expected value of neighbor discovery time by dynamically adjusting the reception probabilities of different sectors, thereby enabling more efficient directional neighbor discovery. El Khamlichi et al. [8,9] propose two novel decentralized, low-complexity reinforcement learning-based directional antenna neighbor discovery schemes, effectively reducing overhead and latency. Wang et al. [10] present a Hunting-based Directional Neighbor Discovery (HDND) scheme for ad hoc mmWave networks, where a node continuously rotates its directional beam to scan the neighborhood, alternately transmitting beacons and listening for acknowledgements (ACKs), implementing spatial rendezvous through deterministic rather than random methods. Sui et al. [11] investigate the application of reinforcement learning algorithms to the directional antenna neighbor discovery process without prior neighbor location information, significantly enhancing neighbor discovery efficiency.

To address the bottleneck of neighbor discovery latency in directional antennas, researchers have proposed advanced prediction-based protocols. For instance, the Neighbor Discovery with Location Prediction protocol (ND-LP) and the Avoiding Communication Interruption with Location Prediction protocol (ACI-LP) [12] are employed to accelerate convergence processes. ND-LP achieves rapid main lobe and channel convergence, while ACI-LP takes it a step further by specializing in beam tracking and channel convergence while actively preventing communication interruptions. Simulation results demonstrate that these protocols outperform existing techniques in reducing neighbor discovery latency. Taking a step further, the authors in [13] adopt a cross-layer optimization approach to realize reliable antenna selection and network maintenance.

In addition, for network optimization under directional communication, He et al. [14] suggest employing a Multi-Agent Deep Distributed Reinforcement Learning (MADDRL) model to adjust directional antennas and node traffic, realizing precise antenna adjustment and interference awareness for adaptive network transmission. Zhai et al. [15] propose a hierarchical beamforming algorithm based on hybrid channel state information (CSI), which effectively balances the trade-off between communication performance and interference suppression requirements. However, this scheme is not inherently designed for the highly-dynamic environments typical of FANETs.

To address the joint optimization of aircraft mobility and communication networks, Hu et al. [16] introduce a Cyber-Physical Routing protocol exploiting Trajectory Dynamics (CPR-TD). By leveraging pre-defined flight trajectory planning information from the application layer as prior knowledge for the network layer, this protocol significantly reduces routing overhead and enhances the Packet Delivery Ratio (PDR) of Mission-Oriented FANETs (MO-FANETs) in complex mission scenarios. Similarly, for FANETs in marching formation, Yang et al. [17] develop a Betweenness Centrality-based Dynamic Source Routing (BC-DSR) protocol. This protocol employs a Gauss-Markov Group (GMG) mobility model to characterize the spatial and temporal correlations of node movements, while utilizing the betweenness centrality metric from graph theory to evaluate node importance, thereby identifying more robust relay paths. Nevertheless, both of these studies are predicated on idealized omnidirectional communication network models.

As shown in Table 1, existing works either (i) improve neighbor discovery/beam training under directional communications without fully modeling 3D attitude-driven installation constraints and FANET-specific dynamics, or (ii) enhance FANETs protocol via mobility/trajectory knowledge but rely on omnidirectional assumptions. This research gap motivates us to develop a constrained antenna selection and beam-pointing control framework for directional FANETs. Specifically, we are the first to explicitly incorporate Guidance, Navigation, and Control (GNC) attitude states (roll/pitch/yaw) together with antenna installation and visibility-domain constraints into the antenna selection and beam-pointing decision process, intending to substantially reduce alignment overhead while maintaining a higher beam alignment success rate under high-speed maneuvering conditions.

### 1.2. Research Contribution

To summarize, most current studies are based on idealized assumptions and do not fully consider the maneuverability of UAVs, altitude differences, and the directionality constraints of directional antennas in three-dimensional space. To the best of our knowledge, no research has explicitly considered the impact of introducing directional antennas under a three-dimensional spatial topology on the network. Therefore, this paper overcomes the limitations brought about by the idealized assumptions prevalent in existing research. It considers antenna selection and beam pointing calculations under a three-dimensional spatial topology, constructs a refined directional communication model for FANETs, and further integrates the dynamic motion characteristics of UAVs with the directionality constraints of antennas to achieve precise antenna selection and beam pointing in highly-dynamic environments. It addresses key issues in the design of communication protocols and algorithms for directional FANETs. The main contributions of this paper are as follows:

A novel joint design paradigm: This study proposes and implements a directional antenna selection and beam pointing control algorithm for FANETs that tightly integrates the communication system with the UAV’s GNC system [18] for the first time. This innovative design, by deeply integrating UAV navigation information with communication requirements, enables real-time adjustment of antenna pointing in complex three-dimensional space, fundamentally enhancing the efficiency and reliability of establishing and maintaining communication links in highly-dynamic environments.

A precise algorithm process based on multi-coordinate system transformation: To address practical engineering issues such as antenna installation location and UAV attitude changes, this study designs a complete transformation algorithm from the Earth-Centered Earth-Fixed (ECEF) coordinate system to the local coordinate system, then to the body coordinate system, and finally to the antenna array coordinate system. This algorithm serves as the mathematical foundation for accurately locating the communication target node and determining the beam pointing, thus effectively addressing the beam pointing problem caused by UAV attitude changes and antenna installation constraints.

Validation of the proposed algorithm through simulation and physical experiment: the results demonstrate that the proposed method can accurately select antennas and determine beam pointing directions, providing fundamental support for the design of directional communication strategies for highly-dynamic UAV clusters.

### 1.3. Paper Organization

The remainder of this paper is organized as follows: Section 2 provides a detailed description of the proposed network model. Section 3 introduces the coordinate system definitions and transformation methods on which the proposed algorithm relies. Section 4 and Section 5 elaborate on the specific implementation process of the antenna selection and beam pointing control algorithm. Section 6 presents the simulation results based on MATLAB R2023b and physical experiment validation, along with an analysis of the results. Finally, Section 7 summarizes the paper and outlines future work directions.

## 2. System Model Description

**Figure 1:**
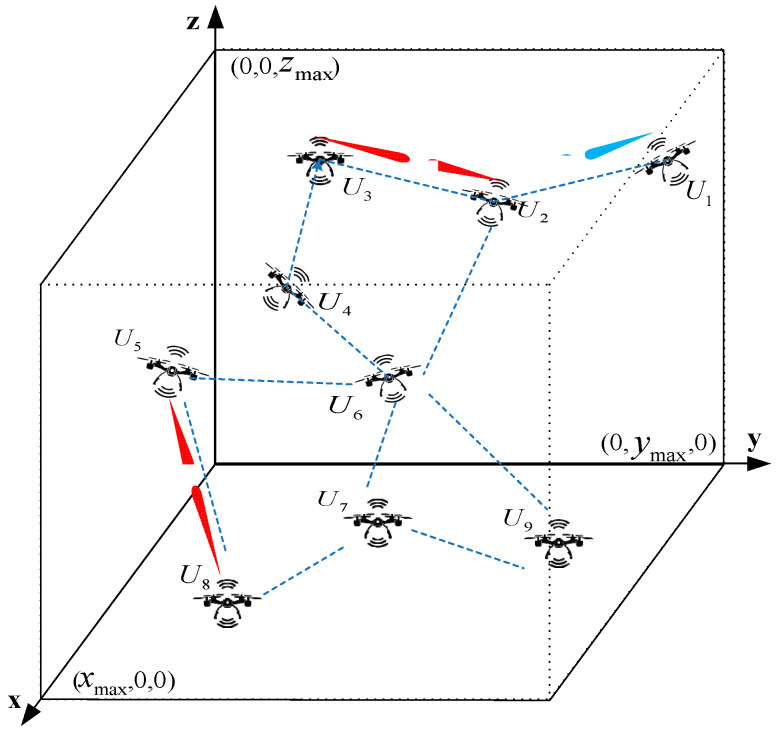
**A** FANET communication scenario.

In this paper, we assume that a FANET consists of no fewer than five nodes, as shown in Figure 1, with each node equipped with five phased array antennas capable of switching between wide and narrow beams (including three around the body’s circumference, one at the front, and one at the rear). The beam angle of the directional antenna should be set according to the capability of the antenna terminal.

Additionally, the network possesses the following characteristics:

The aerial nodes are the primary components of the FANET, enabling direct communication between aircraft, with ground nodes and communication relay nodes not considered in this study.

The aerial nodes exhibit high mobility, resulting in rapid changes in network topology [19].

The directional communication range between aircraft is considerable, and it is assumed that all communication nodes are within effective communication range.

It is assumed that each aircraft is equipped with positioning systems such as a Global Positioning System (GPS) [20] and an Inertial Measurement Unit (IMU) [21], which can acquire its pose information within a short time and obtain the position information of neighboring nodes through network maintenance messages.

It is assumed that before conducting directional antenna-aided networking [22], the nodes in the FANETs have completed network formation via wide-beam scanning and have preliminary shared location information.

**Figure 2:**
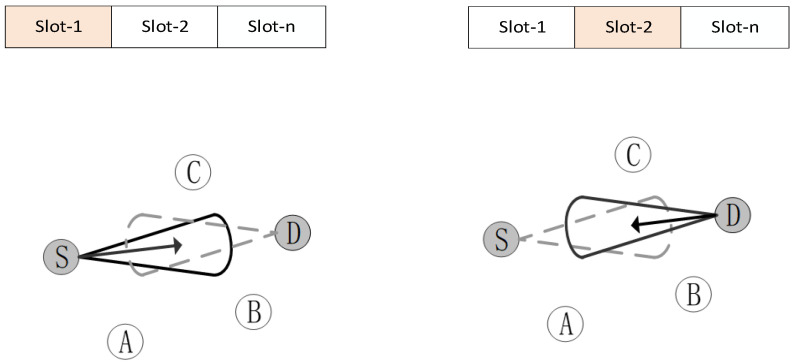
Directional networking and chain-building process.

According to the networking protocol, the two communication nodes can exchange information within the same time slot [23]. It requires one node to be receiving while the other is transmitting, as shown in Figure 2. Through antenna selection and beamforming algorithms, their transmitting and receiving beams can be made to overlap.

## 3. Definition of Common Coordinate Systems and Coordinate System Transformation

### 3.1. Coordinate System Definitions

#### 3.1.1. ECEF Rectangular Coordinate System (Abbreviated as E System)

The origin of the coordinate system is located at the Earth’s center. The X_e_-axis lies in the Earth’s equatorial plane and points toward the Greenwich meridian (zero meridian). The Z_e_-axis is perpendicular to the equatorial plane, aligned with the Earth’s rotation axis, and points toward the North Pole. It forms a right-handed rectangular coordinate system. Given that the Earth is an ellipsoid, there are multiple standards [24] for defining coordinates. This paper adopts the CGCS2000 Earth model parameters [25].

#### 3.1.2. Local North-East-Up Coordinate System (Abbreviated as T System)

The origin of the coordinate system can be selected as needed [26]. The Y_t_-axis is aligned with the vector from the Earth’s center to the origin. The X_t_-axis lies in the local meridian plane, is perpendicular to the Y_t_-axis, and points north. The O-X_t_Y_t_Z_t_ forms a right-handed rectangular coordinate system.

#### 3.1.3. Aircraft Body Coordinate System (Abbreviated as B System)

**Figure 3:**
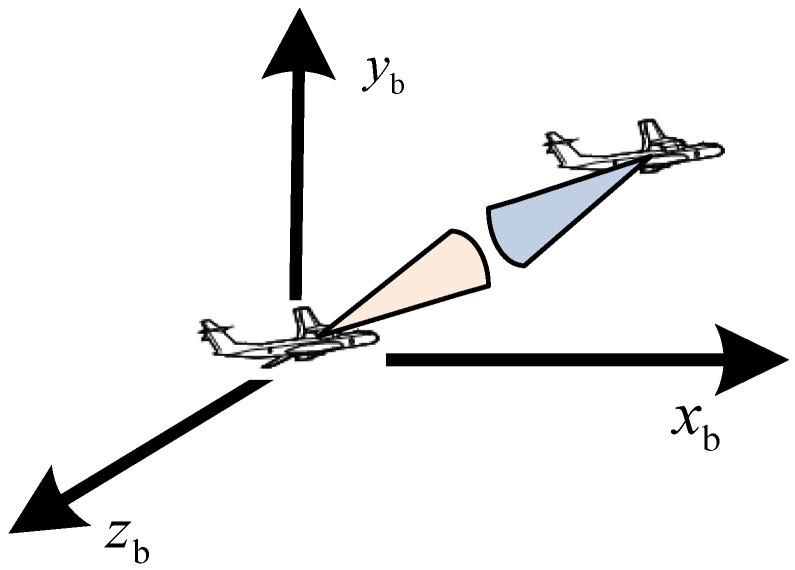
Aircraft body coordinate system.

The coordinate system originates from the center of mass of the aircraft [27]. The X_b_-axis aligns with the longitudinal symmetry axis of the aircraft and extends toward the tip of the nose. The Y_b_-axis lies in the longitudinal symmetry plane, is perpendicular to the X_b_-axis, and points upwards [28]. These three axes together form a right-handed rectangular coordinate system, as shown in Figure 3.

#### 3.1.4. Antenna Array Coordinate System (Abbreviated as A System)

The antenna coordinate system is defined by considering the normal direction of the phased array antenna as the *Z*-axis, designating the initial direction of rotation of the antenna array plane as the *X*-axis [29], and identifying the direction perpendicular to the *X*-axis within the array plane as the *Y*-axis. Collectively, these three axes establish a right-handed Cartesian coordinate system.

### 3.2. Coordinate System Transformation

The coordinate system transformation definitions are shown in Table 2.

#### 3.2.1. General Formula for Coordinate Transformation

We present the following definitions:

(1) $$M_{1}(\alpha )=\left[\begin{array}{ccc} 1 & 0 & 0\\ 0 & \cos\alpha & \sin\alpha \\ 0 & -\sin\alpha & \cos\alpha \end{array}\right]$$

(2) $$M_{2}(\alpha )=\left[\begin{array}{ccc} \cos\alpha & 0 & -\sin\alpha \\ 0 & 1 & 0\\ \sin\alpha & 0 & \cos\alpha \end{array}\right]$$

(3) $$M_{3}(\alpha )=\left[\begin{array}{ccc} \cos\alpha & \sin\alpha & 0\\ -\sin\alpha & \cos\alpha & 0\\ 0 & 0 & 1 \end{array}\right]$$

where α is the rotation angle around the coordinate axis.

#### 3.2.2. E System to T System

Assuming the origin of the T system is at a location with longitude *L* and latitude *B* (positive for north latitude, negative for south latitude), the E system can be transformed into the T system through three steps:

Step 1: Rotate the E system clockwise around the Z_e_-axis by ($\frac{\pi }{2}-L$) to obtain the first rotation matrix $M_{3}(-\frac{\pi }{2}+L)$. At this point, the new OY_e_ is in the same meridian plane as the OY_t_ of the T system.

Step 2: Rotate the new OX_e_ axis counterclockwise by the geographic latitude *B* to obtain the second rotation matrix $M_{1}(B)$. At this point, the new OY_e_ axis is parallel to the OY_t_ axis of the T system, and the new OZ_e_ axis is parallel to the OX_t_ axis.

Step 3: Rotate the new OY_e_ axis clockwise by $\frac{\pi }{2}$ to obtain the third rotation matrix $M_{2}(-\frac{\pi }{2})$.

Thus, the coordinate transformation matrix [30] from the E system to the T system is:

(4) $$C_{E}^{T}=M_{2}(-\frac{\pi }{2})M_{1}(B)M_{3}(-\frac{\pi }{2}+L)$$

#### 3.2.3. T System to B System

The attitude angles of the body are defined with respect to the T system as follows:

- Pitch angle: The angle between the projection of the aircraft’s longitudinal axis on the horizontal plane of the T system and the aircraft’s longitudinal axis is defined as the pitch angle. It is positive when upward and negative when downward.
- Yaw angle: The angle between the projection of the aircraft’s longitudinal axis on the horizontal plane of the T system and the *X*-axis direction of the launch coordinate system.
- Roll angle: The angle between the aircraft’s transverse axis and the horizontal plane of the T System [31]. The transformation matrix from the T system to the B system, after three rotations, is given by:

(5) $$\begin{array}{l} C_{T}^{B}=M_{1}(\gamma )M_{3}(\theta )M_{2}(\psi )\\ =\left[\begin{array}{ccc} 1 & 0 & 0\\ 0 & \cos\gamma & \sin\gamma \\ 0 & -\sin\gamma & \cos\gamma \end{array}\right]\left[\begin{array}{ccc} \cos\theta & \sin\theta & 0\\ -\sin\theta & \cos\theta & 0\\ 0 & 0 & 1 \end{array}\right]\left[\begin{array}{ccc} \cos\psi & 0 & -\sin\psi \\ 0 & 1 & 0\\ \sin\psi & 0 & \cos\psi \end{array}\right]\\ =\left[\begin{array}{ccc} \cos\theta \cos\psi & \sin\theta & -\cos\theta \sin\psi \\ -\sin\theta \cos\psi \cos\gamma +\sin\psi \sin\gamma & \cos\theta \cos\gamma & \sin\theta \sin\psi \cos\gamma +\cos\psi \sin\gamma \\ \sin\theta \cos\psi \sin\gamma +\sin\psi \cos\gamma & -\cos\theta \sin\gamma & -\sin\theta \sin\psi \sin\gamma +\cos\psi \cos\gamma \end{array}\right] \end{array}$$

## 4. Antenna Installation Position and Coordinate System Definition

### 4.1. Antenna Installation Position

**Figure 4:**
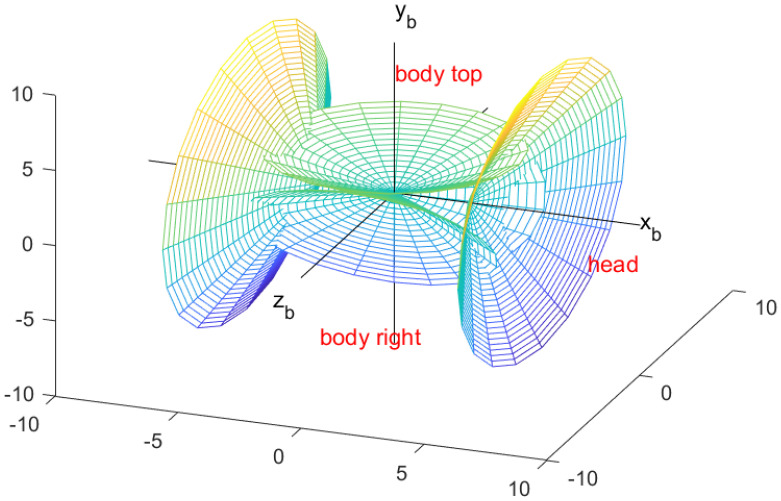
Simulated illustration of the signal radiation with different antenna installation positions.

The antennas are mounted on the exterior structure of the aircraft, specifically one set at the nose, three sets along the fuselage, and one set at the tail, to ensure comprehensive and stable signal coverage, as shown in Figure 4. In an ideal scenario, when two aircraft are engaged in directional antenna-based network communication, the center of gravity of the aircraft is designated as the coordinate origin [32].

The straight line defined by the rotational axes of the antennas located at the nose and tail coincides with the aircraft’s X_b_-axis [33], and the opening direction of the nose antenna is aligned with the positive direction of the X_b_-axis. The normal to the antenna located on the top of the fuselage is aligned with the Y_b_-axis of the aircraft, and the normal to the antennas on the left and right sidewalls form a 60-degree angle with the plane of the aircraft’s longitudinal axis.

### 4.2. B System to A System

Body Top: Taking the center of gravity of the carrier as the vertex and the vector ***b***_1_ = (0,1,0) of the B system as the normal Z-axis of the antenna, the inner space of the cone at the bottom of infinity is divided into the responsible area of the body top antenna [34]. When the communication target node is located in this space, the body top antenna is in operation, and the other antennas are disabled. The transformation matrix from the B system to the body top antenna array coordinate system is calculated as follows:

(6) $$C_{B}^{A}(top)=M_{1}(-\frac{\pi }{2})$$

If the antenna has an installation deviation, such as an angle of $a_{1}$ radians that rotates counterclockwise around the X_b_-axis of the aircraft body, the corresponding antenna normal vector and transformation matrix are calculated as follows:

(7) $$b_{1}=(0,\;sin(\frac{\pi }{2}+a_{1}),\;cos(\frac{\pi }{2}+a_{1}))$$

(8) $$C_{B}^{A}(top)=M_{1}(-\frac{\pi }{2}+\alpha_{1})$$

Body Left: The circumferential antenna with the vector ***b***_2_$\;=\left(0,-\frac{1}{2},-\frac{\sqrt{3}}{2}\right)$ located in the I quadrant of the B system as its normal vector is the body left antenna. The transformation matrix from the B system to the body left antenna array coordinate system is calculated as follows:

(9) $$C_{B}^{A}(left)=M_{1}(-\frac{7\pi }{6})$$

If the antenna has an installation deviation, such as an angle of $a_{2}$ radians that rotates counterclockwise around the X_b_-axis of the aircraft body, the corresponding antenna normal vector and transformation matrix are as follows:

(10) $$b_{2}=(0,-\sin(\frac{\pi }{6}+a_{2}),-\cos(\frac{\pi }{6}+a_{2}))$$

(11) $$C_{B}^{A}(left)=M_{1}(-\frac{7\pi }{6}+\alpha_{2})$$

Body Right: The circumferential antenna with the vector $\left(0,-\frac{1}{2},\frac{\sqrt{3}}{2}\right)$ located in the IV quadrant of the B system as its normal vector is the body right antenna. The transformation matrix from the B system to the body right antenna array coordinate system is calculated as follows:

(12) $$C_{B}^{A}(right)=M_{1}(\frac{\pi }{6})$$

If the antenna has an installation deviation, such as $a_{3}$ radians that rotates counterclockwise around the X_b_-axis of the aircraft body, the corresponding antenna normal vector and transformation matrix are as follows:

(13) $$b_{3}=(0,\sin(-\frac{\pi }{6}+a_{3}),\cos(-\frac{\pi }{6}+a_{3}))$$

(14) $$C_{B}^{A}(right)=M_{1}(\frac{\pi }{6}+\alpha_{3})$$

Head: The front antenna, referred to as the nose antenna, has the normal vector aligned with the vector (1, 0, 0) in the B system. The transformation matrix from the B system to the head antenna array coordinate system is given as follows:

(15) $$C_{B}^{A}(head)=M_{2}(\frac{\pi }{2})\;M_{1}(-\frac{\pi }{2})$$

Tail: The rear antenna, referred to as the tail antenna, has the normal vector aligned with the vector (−1, 0, 0) in the B system. The transformation matrix from the B system to the tail antenna array coordinate system is given as follows:

(16) $$C_{B}^{A}(tail)=M_{2}(-\frac{\pi }{2})\;M_{1}(-\frac{\pi }{2})$$

### 4.3. Transformation from Phased Array Antenna Coordinate System to Off-Axis Angle and Rotation Angle

**Figure 5:**
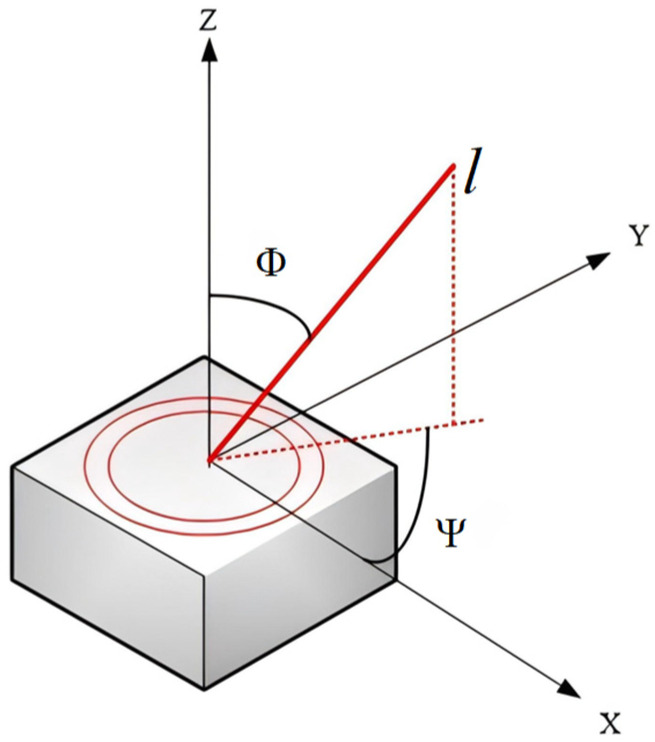
Schematic diagram of the antenna array coordinate system.

The transformation from the phased array antenna coordinate system to the off-axis angle Φ and rotation angle Ψ can be derived based on the definitions provided in [35], as shown in Figure 5. Then we have

(17) $$\left[\begin{array}{c} l\cdot \sin\Phi \cdot \cos\Psi \;\;\;\\ l\cdot \sin\Phi \cdot \sin\Psi \;\;\;\\ l\cdot \cos\Phi \end{array}\right]=\left[\begin{array}{c} \Delta x^{\left(A\right)}\\ \Delta y^{\left(A\right)}\\ \Delta z^{\left(A\right)} \end{array}\right]$$

(18) $$\begin{array}{l} l=\sqrt{{\left(\Delta x^{\left(A\right)}\right)}^{2}+{\left(\Delta y^{\left(A\right)}\right)}^{2}+{\left(\Delta z^{\left(A\right)}\right)}^{2}}\\ \Psi =\tan^{-1}(\frac{\Delta y^{\left(A\right)}}{\Delta x^{\left(A\right)}}),\;\;\;\;\;(-\pi \leq \Psi \leq \pi )\\ \Phi =\cos^{-1}(\frac{\Delta z^{\left(A\right)}}{l}),\;\;\;\;\;(0\leq \Phi \leq \frac{\pi }{2}) \end{array}$$

where $\left[\Delta x^{\left(A\right)},\Delta y^{\left(A\right)},\Delta z^{\left(A\right)}\right]$ represents the distance vector between two nodes in the antenna coordinate system.

## 5. Antenna Selection and Beam Direction Control Algorithm and Simulation Verification

### 5.1. Antenna Selection Algorithm

**Figure 6:**
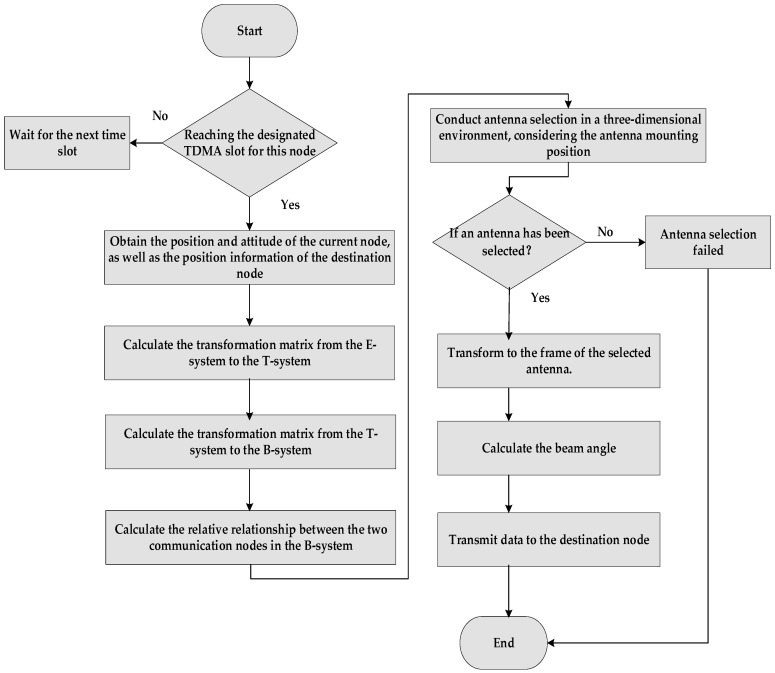
Flowchart of the proposed algorithm.

The main processes of the proposed algorithm include input parameter analysis, coordinate transformation, translation vector calculation, and antenna coverage angle calculation, as shown in Figure 6. The steps of the proposed algorithm are explained as follows.

1. Input the position coordinates of the communication source node in the E system (*x_i_, y_i_, z_i_*), the position of the communication target node (*x_j_, y_j_, z_j_*), and the attitude information of the communication source node.
2. Coordinate conversion ①: Transform the coordinates of both nodes from the E system to the T system using the transformation matrix.
  (19) $$\left[\begin{array}{c} x_{i}^{T}\\ y_{i}^{T}\\ z_{i}^{T} \end{array}\right]=C_{E}^{T}\cdot \left[\begin{array}{c} x_{i}\\ y_{i}\\ z_{i} \end{array}\right],\;\left[\begin{array}{c} x_{j}^{T}\\ y_{j}^{T}\\ z_{j}^{T} \end{array}\right]=C_{E}^{T}\cdot \left[\begin{array}{c} x_{j}\\ y_{j}\\ z_{j} \end{array}\right]$$
3. Coordinate conversion ②: Further transform the coordinates obtained in Step 2 to the body coordinate system.
  (20) $$\left[\begin{array}{c} x_{i}^{\left(B\right)}\\ y_{i}^{\left(B\right)}\\ z_{i}^{\left(B\right)} \end{array}\right]=C_{T}^{B}\cdot \left[\begin{array}{c} x_{i}^{\left(T\right)}\\ y_{i}^{\left(T\right)}\\ z_{i}^{\left(T\right)} \end{array}\right],\;\left[\begin{array}{c} x_{j}^{\left(B\right)}\\ y_{j}^{\left(B\right)}\\ z_{j}^{\left(B\right)} \end{array}\right]=C_{T}^{B}\cdot \left[\begin{array}{c} x_{j}^{\left(T\right)}\\ y_{j}^{\left(T\right)}\\ z_{j}^{\left(T\right)} \end{array}\right]$$
4. Translation vector calculation: Compute the translation vector in the B System.
  (21) $$\vec{a}=\left[\begin{array}{c} \Delta x^{\left(B\right)}\\ \Delta y^{\left(B\right)}\\ \Delta z^{\left(B\right)} \end{array}\right]=\left[\begin{array}{c} x_{j}^{\left(B\right)}\\ y_{j}^{\left(B\right)}\\ z_{j}^{\left(B\right)} \end{array}\right]-\left[\begin{array}{c} x_{i}^{\left(B\right)}\\ y_{i}^{\left(B\right)}\\ z_{i}^{\left(B\right)} \end{array}\right]$$
5. Calculate the cosine of the angle between the communication target vector and all normal antennas:
  (22) $$\cos<\vec{a},\vec{b}>=\frac{\vec{a}•\vec{b}}{\left|\vec{a}|•|\vec{b}\right|}$$
  where $\vec{b}$ is the direction vector of the normal antenna and $\vec{a}$ is the coordinate vector of the communication target node.
6. Selection: Choose the direction vector that maximizes the value obtained in Step 5. The corresponding antenna is the selected antenna.

### 5.2. Beam Direction Control Algorithm

The beam direction control algorithm builds upon the antenna selection algorithm by further transforming the coordinates and calculating the off-axis angle and rotation angle of the translation vector in the antenna coordinate system to determine the antenna’s direction. More specifically, after finishing Steps 1–6 of the antenna selection algorithm, the following steps are executed.

1. Coordinate conversion ③: Transform the coordinates of the body system calculated in Step 3 of the antenna selection algorithm to the antenna coordinate system, based on the antenna selected in the above Step 6.
  (23) $$\left[\begin{array}{c} x_{i}^{\left(A\right)}\\ y_{i}^{\left(A\right)}\\ z_{i}^{\left(A\right)} \end{array}\right]=C_{B}^{A}\cdot \left[\begin{array}{c} x_{i}^{\left(B\right)}\\ y_{i}^{\left(B\right)}\\ z_{i}^{\left(B\right)} \end{array}\right],\;\left[\begin{array}{c} x_{j}^{\left(A\right)}\\ y_{j}^{\left(A\right)}\\ z_{j}^{\left(A\right)} \end{array}\right]=C_{B}^{A}\cdot \left[\begin{array}{c} x_{j}^{\left(B\right)}\\ y_{j}^{\left(B\right)}\\ z_{j}^{\left(B\right)} \end{array}\right]$$
2. Translation vector calculation: Compute the translation vector in the A System.
  (24) $$\vec{c}=\left[\begin{array}{c} \Delta x^{\left(A\right)}\\ \Delta y^{\left(A\right)}\\ \Delta z^{\left(A\right)} \end{array}\right]=\left[\begin{array}{c} x_{j}^{\left(A\right)}\\ y_{j}^{\left(A\right)}\\ z_{j}^{\left(A\right)} \end{array}\right]-\left[\begin{array}{c} x_{i}^{\left(A\right)}\\ y_{i}^{\left(A\right)}\\ z_{i}^{\left(A\right)} \end{array}\right]$$
3. Off-axis and rotation angle calculation: Calculate the off-axis angle and rotation angle of the vector in the antenna coordinate system as described in Section 4.3.

### 5.3. Complexity Analysis of the Proposed Algorithm

The core of the proposed algorithm involves coordinate system transformations (matrix multiplications). Meanwhile, we implement the Time Division Multiple Access (TDMA) protocol at the MAC layer, ensuring that a node in a large-scale distributed network can only communicate with one other node per time slot. For a single neighbor, the process involves a fixed sequence of 3×3 matrix multiplications and trigonometric calculations. This is an O(1) operation with a very small constant factor. Given that for modern embedded processors on UAVs, calculating these transformations for even high-density scenarios takes negligible time (on the order of microseconds), this approach is more suitable for unmanned platform deployment than the iterative optimization algorithms or deep learning algorithms commonly used in other solutions.

## 6. Simulation Results and Analysis

### 6.1. Simulation

**Figure 7:**
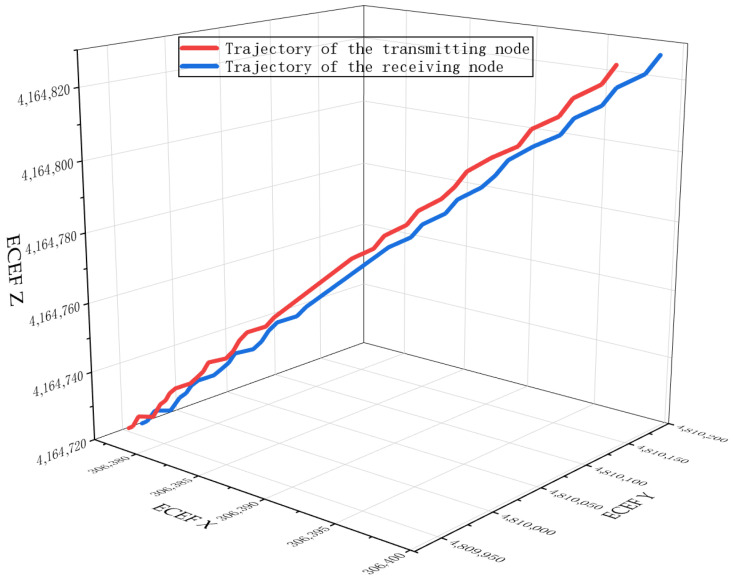
Aircraft trajectory.

To verify the effectiveness of the proposed algorithm, simulation experiments were conducted with MATLAB R2023b. The goal was to verify the correctness of the proposed algorithm and evaluate its performance by comparing it with traditional algorithms. In the experiments, the position and attitude information of multiple aircraft nodes were used as input, with the maximum transmission distance between the two nodes set to 10 km. The simulation experiments employ a TDMA network with *N* nodes. Each node is equipped with distinct trajectory data to simulate aircraft maneuvers, as shown in Figure 7. To capture the highly-dynamic property of nodes, the maximum node speed was set to 500 m/s. Given that nodes in FANETs typically operate in open airspace where links are more likely to exhibit Line-of-Sight (LoS) propagation, and that the free-space assumption is widely adopted in related studies [3,36], this work employs the Friis free-space path-loss model to characterize large-scale fading. Other experimental parameters are listed in Table 3.

#### 6.1.1. Experiment 1: Algorithm Selection Results Under Fixed Position and Attitude Information

**Figure 8:**
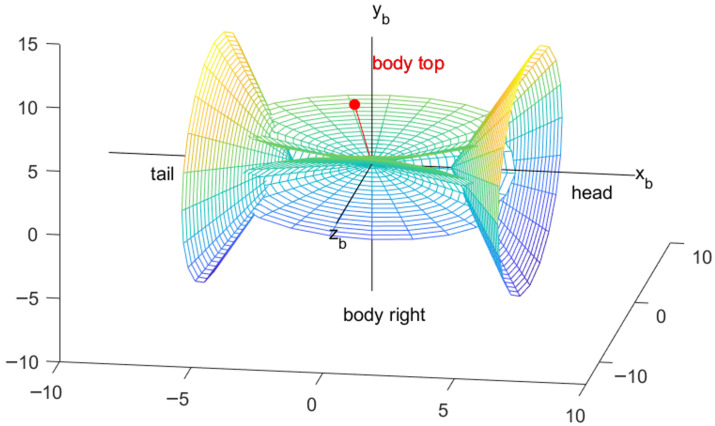
Simulation result of selecting the top antenna. The red dot represents the communication target node, and the communication source node is at the origin.

**Figure 9:**
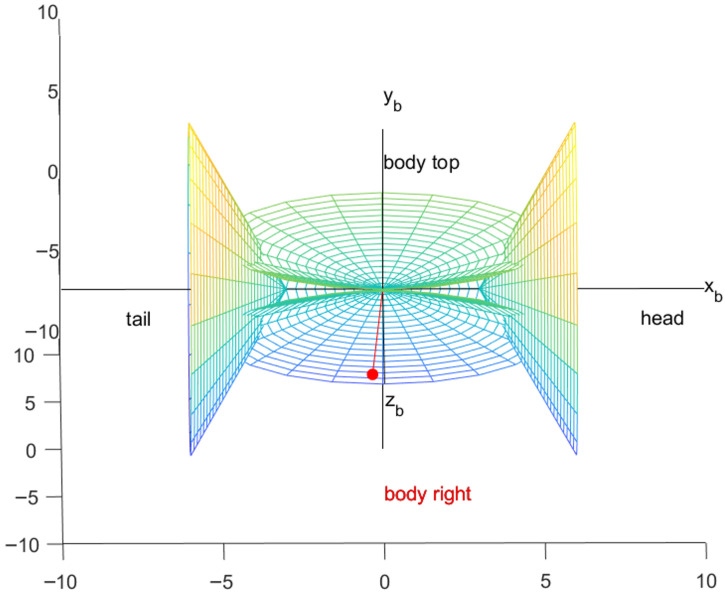
Simulation result of selecting the right antenna. The red dot represents the communication target node, and the communication source node is at the origin.

**Figure 10:**
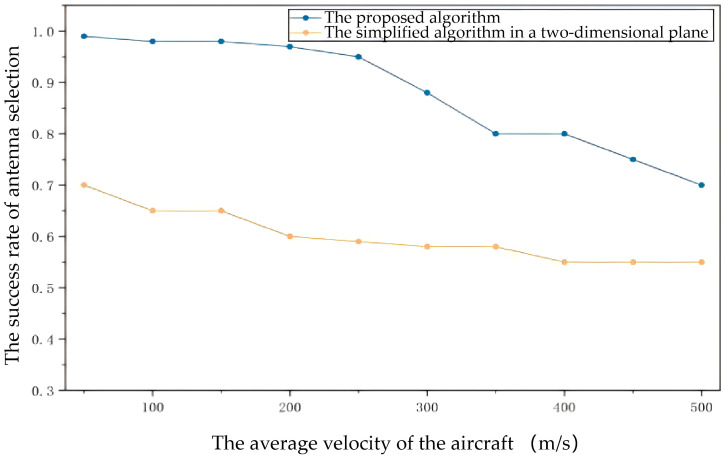
The success rate of antenna selection of the proposed algorithm and the simplified algorithm in a two-dimensional plane.

The simulation results for antenna selection are shown in Figure 8 and Figure 9, and the simulation results of the success rate of antenna selection are shown in Figure 10. We can see that the proposed algorithm accurately selects the appropriate antenna and determines the off-axis and rotation angles. The probability of correct selection is as high as 98%, meeting the requirements of practical applications.

#### 6.1.2. Experiment 2: Adaptability in High-Mobility Scenarios

**Figure 11:**
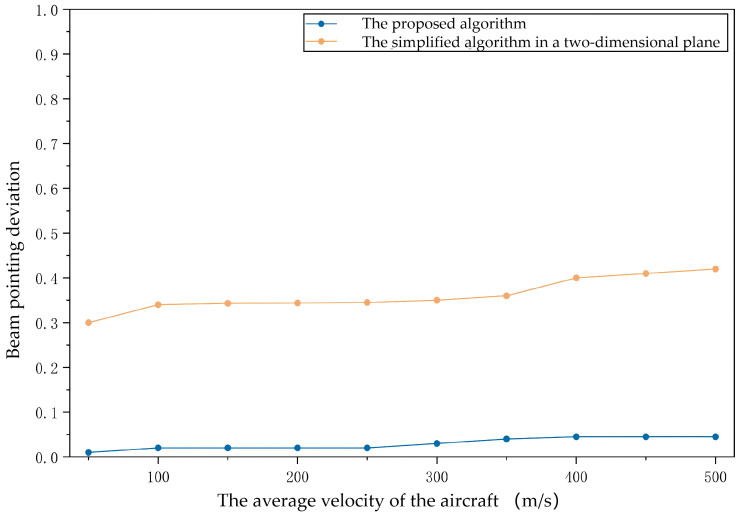
Beam pointing deviation of the proposed algorithm and the simplified algorithm in a two-dimensional plane.

To further verify the applicability of the proposed algorithm in highly-dynamic environments, multiple sets of comparative experiments were conducted under different flight speed conditions to evaluate the performance differences between the proposed algorithm and the traditional methods in highly-dynamic network environments. The experimental results shown in Figure 10 and Figure 11 indicate that as the flight speed increases, the proposed algorithm can maintain a high antenna selection accuracy and beam pointing accuracy. Compared with the simplified algorithm in a two-dimensional plane (note that the simplified algorithm is essentially a degraded version of the proposed algorithm without considering attitude constraints when performing calculations), the proposed algorithm demonstrates superior adaptability and robustness. The benchmarking method’s accuracy drops sharply as speed increases (dropping to below 70% at 200 m/s) because it fails to account for the rapid pitch and roll changes of the UAVs during highly-dynamic maneuvers. Our algorithm utilizes real-time attitude data from the GNC system to compensate for these changes, maintaining over 95% accuracy.

Meanwhile, the beam pointing deviation of the proposed algorithm consistently maintains a low level, significantly outperforming traditional algorithms. These results fully demonstrate that the proposed algorithm in this paper has stronger adaptability and higher reliability in highly-dynamic scenarios and is better able to meet the requirements for communication performance in practical applications.

It is important to clarify the rationale for selecting the “Simplified Algorithm in a Two-Dimensional Plane” as the comparison baseline. While in Section 1.1 we have discussed various advanced directional protocols (e.g., reinforcement learning-based [8,9] or prediction-based [12] methods), a direct quantitative comparison with these specific protocols is challenging due to the unique assumption of this paper: the tight integration of GNC data for real-time correction. Most existing studies rely on simplified point-mass models or assume idealized antenna stability, neglecting the rapid attitude changes (roll, pitch, and yaw) inherent in highly-dynamic maneuvers.

In contrast, the “Simplified Algorithm in a Two-Dimensional Plane” effectively represents the performance ceiling of conventional topology-based methods that rely solely on position information while ignoring attitude constraints. As shown in Figure 10 and Figure 11, the significant performance divergence at high speeds (e.g., >200 m/s) quantitatively demonstrates the necessity of the proposed 3D coordinate transformation strategy. The results confirm that in highly-dynamic FANETs, simply knowing the 3D position is insufficient; real-time attitude compensation via GNC integration is critical for maintaining stable directional links.

#### 6.1.3. Experiment 3: The Influence of Positioning and Attitude Estimation Deviation on the Proposed Algorithm

**Figure 12:**
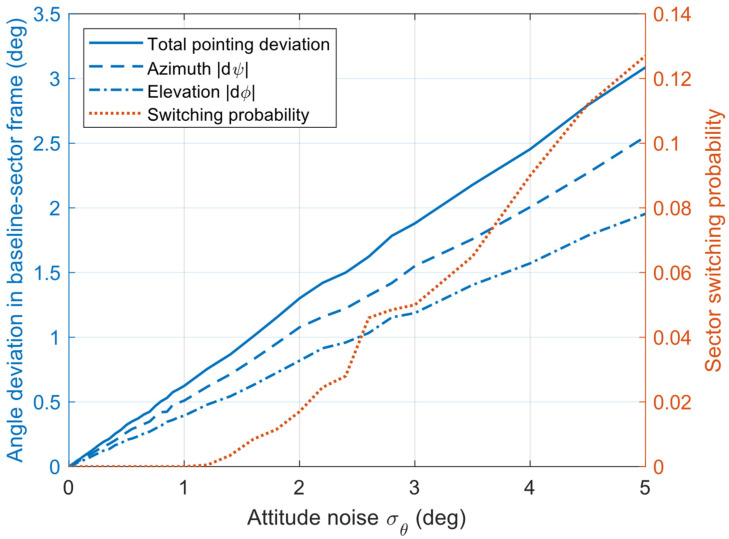
The influence of attitude deviation on the proposed algorithm.

**Figure 13:**
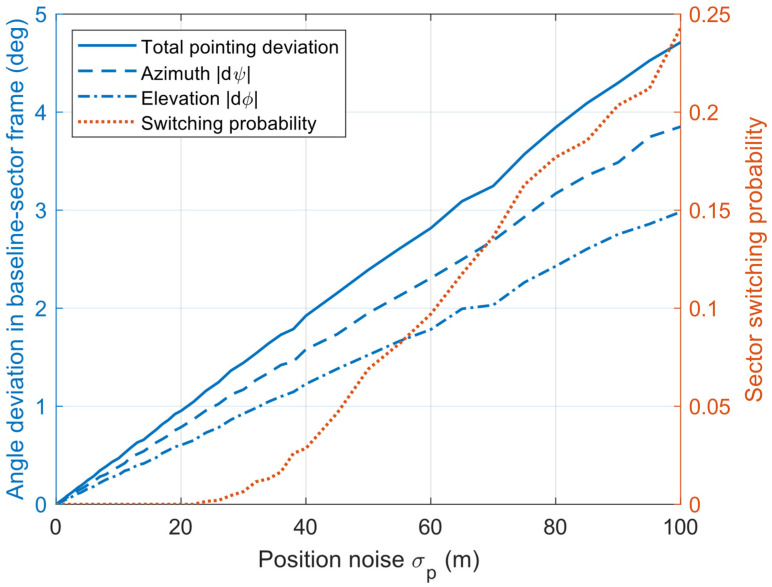
The influence of position deviation on the proposed algorithm.

The experiments inject additive Gaussian white noise into the current node position and attitude measurements to emulate practical estimation errors. Given the input trajectories and a relative inter-node distance not exceeding 3 km, the simulation results are shown in Figure 12 and Figure 13. The impact of attitude Gaussian noise on pointing accuracy is relatively mild: when $\sigma_{\theta }$ increases to 5°, the total pointing deviation is approximately 3.1°, while the antenna switching probability remains only about 0.12, indicating that the dominant effect manifests as continuous angular perturbations. Likewise, the Gaussian noise of position has a limited influence on beam pointing and antenna-selection stability. When $\sigma_{Ρ}$ increases to 100 m, the total pointing deviation rises to about 4.5°, and the sector switching probability increases to approximately 0.22, suggesting that meter-to-hectometer-level position perturbations under the considered geometric configuration (i.e., relatively small inter-node separation) can still maintain low pointing errors and a low switching probability. Overall, these results demonstrate that, in directional FANETs, position and attitude estimation errors exert a relatively limited impact on antenna selection and beam pointing, and this impact is expected to further diminish as the inter-node distance increases.

#### 6.1.4. Experiment 4: Performance Evaluation

**Figure 14:**
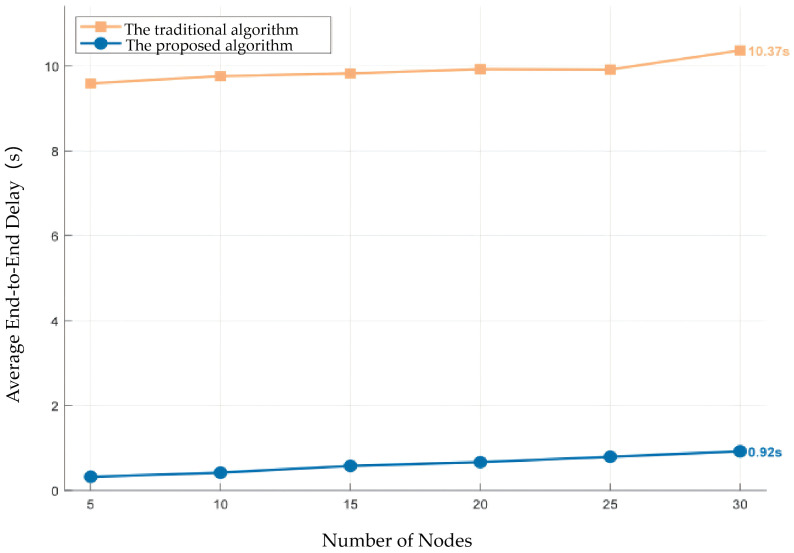
Average E2E latency statistics of different network sizes for the proposed algorithm and the traditional sequential scanning algorithm of [37].

The next experiment compares E2E delay and PDR. Figure 14 plots the average delay versus the number of nodes under TDMA. The End-to-End (E2E) delay is measured from packet generation at the source to successful reception at the destination, and it reflects MAC access waiting, beam alignment, and transmission/re-transmission time. As *N* increases from 5 to 30, the traditional sequential scanning method [37] exhibits a dramatic rise in delay, exceeding 10 s on average; the proposed algorithm maintains a delay of around 0.92 s. The large disparity stems from the scanning overhead that scales linearly with both the number of beams and the number of nodes in the sequential method. In contrast, the proposed algorithm avoids exhaustive search by using estimated position and attitude to compute the azimuth of the communication target node. Even when position estimation errors occur, restricting the search to a local window vastly reduces alignment time and retains high success probability. Consequently, the proposed method achieves much lower latency.

**Figure 15:**
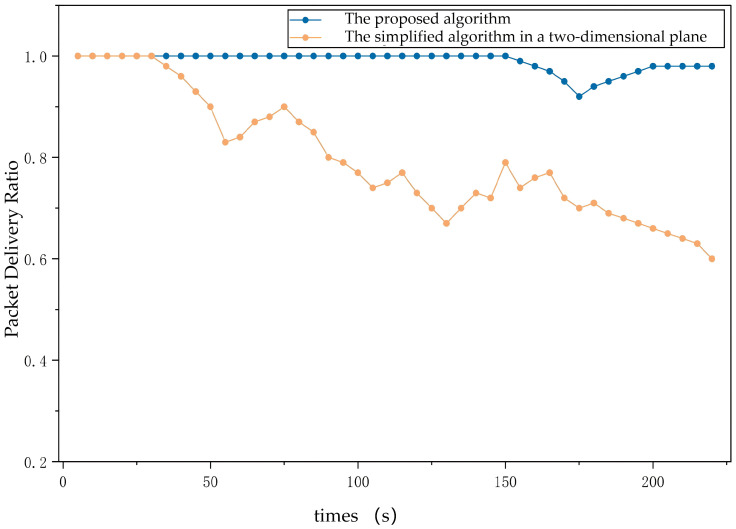
PDR comparison of the proposed algorithm and the simplified algorithm in a two-dimensional plane.

For the PDR, the proposed algorithm also outperforms the baseline because rapid alignment reduces queueing delays and packet drops. Figure 15 demonstrates that the position-based scheme achieves a PDR close to unity across all network sizes, whereas the traditional method suffers from decreasing PDR due to longer idle periods waiting for beam alignment.

### 6.2. Physical Experiment

**Figure 16:**
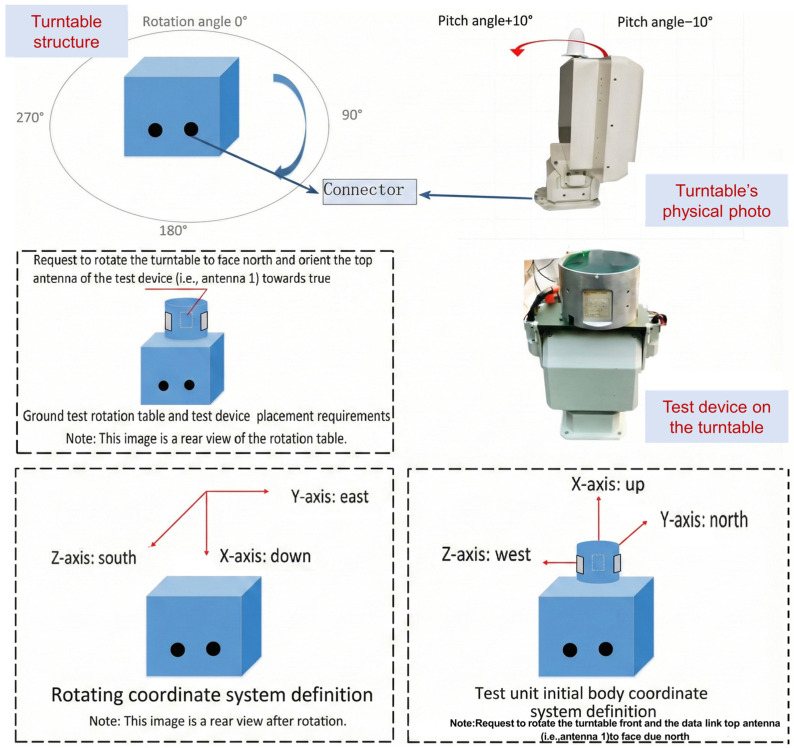
System components and schematic diagram of the physical experiment.

To further verify the effectiveness of the proposed algorithm, the proposed algorithm was ported to a physical system. The experiment consists of two cylindrical test devices A and B (each equipped with three antennas mounted circumferentially), two turntables that can rotate and pitch, and a debugging computer. The test devices are fixed on the turntables, with specific coordinate system definitions and placement requirements as shown in Figure 16.

The specific experimental design is as follows:

1. During the test, one turntable remains stationary with test device A, while the other turntable with test device B rotates, testing the antenna selection and beam calculation for A and B.
2. During the test, one turntable remains stationary with test device A, while the other turntable with test device B pitches, testing the antenna selection and beam calculation for A and B.
3. During the test, one turntable remains stationary with test device A, while the other turntable with test device B first rotates and then pitches, testing the antenna selection and beam calculation for A and B.

The test results in Table 4 indicate that as the turntable rotates, the antenna selected by device B also changes. When rotated to 180 degrees, the antenna selected by device B matches that of device A. If the turntable only pitches without rotation (limited by the turntable, the pitch angle does not exceed 60 degrees), the antenna selected by device B does not change, but the beam angle changes. When device B is simultaneously rotated and pitched, both the antenna selection and beam calculation results change. During the test, the two test devices achieved normal communication through the selected antennas and calculated beam pointing results. Additionally, it was determined that the selected antennas and calculated beam angles are consistent with expectations when combined with the actual deployment location.

Moreover, in multiple repeated experiments, although there is a certain probability of failure in antenna selection, the communication quality remains stable, with a packet delivery rate reaching 100%. Further analysis reveals that during the antenna selection process, coverage blind spots may occur due to changes in the aircraft’s attitude angle. When the calculated off-axis angle is very close to the critical value, it is determined as an antenna selection failure in the simulation. However, in the actual terminal communication process, communication can still be achieved through the gain of the side lobes. It is worth noting that we also evaluated the communication performance of the device in an interference environment by introducing an additional single-direction jammer on top of Test 3. The results show that high directional gain can effectively suppress interference, while accurate beam pointing is crucial for overcoming such channel impairments.

It should be noted that the current antenna installation is designed for simplified scenarios. In practice, the drone platform will face additional challenges, such as structural occlusion (shadow effect) caused by wings/tail fins and engine vibrations, which must be incorporated into future field tests.

## 7. Conclusions and Future Work

### 7.1. Summary of Contributions

This paper addresses the critical challenge of establishing high-efficiency and high-reliability communication links in aircraft clusters under directional connectivity constraints. Moving beyond traditional methods that treat communication and flight control as isolated subsystems, this study demonstrates that the deep integration of the GNC system with the communication system is a feasible optimization scheme.

### 7.2. Key Findings

By mapping the maneuvering characteristics of aircraft in three-dimensional space directly to the physical constraints of antenna directivity, we proposed a deterministic beam pointing and antenna selection framework. This approach shifts the paradigm from “reactive scanning” (which suffers from high latency) to “proactive calculation” (which utilizes navigation state for instant alignment). This approach enables real-time adjustment of antenna selection and beam pointing in highly-dynamic environments, significantly enhancing the efficiency of establishing and maintaining communication links. It provides an effective solution for high-reliability directional networking of aircraft groups under long-range, highly-dynamic directional connection constraints.

Simulation and physical experiment results indicate that the proposed method can accurately calculate the required antenna selection and beam pointing angles based on the attitude information of the flying nodes. The performance of this method is superior to simplified algorithms based on two-dimensional plane assumptions, particularly in its ability to maintain stable links during high-speed maneuvers and complex attitude changes. The results confirm that utilizing GNC data can reduce alignment latency by an order of magnitude compared to traditional scanning methods, providing a robust physical layer foundation for upper-layer networking.

### 7.3. Future Work

For more specific application scenarios, further optimization of the current modeling and calculations is still required. Future research should focus on the impact of the non-coincidence of the antenna array center and the inertial navigation center. It should also address the potential issue of position information loss during high-speed flight by designing fault-tolerant beam control strategies. Additionally, the influence of aircraft navigation accuracy on the proposed algorithm should be considered, and the dynamic adjustment algorithm for antenna pointing should be optimized to enhance the system’s robustness and adaptability. Moreover, complex factors in actual flight environments, such as electromagnetic interference and signal blockage, need to be thoroughly investigated in subsequent work. Furthermore, the co-optimization of multi-antenna spatial multiplexing and multi-hop routing mechanisms should be explored to further improve the scalability and operational efficiency of aircraft group networking while meeting the real-time and reliability requirements of communication in highly-dynamic environments.

## Figures and Tables

**Table 1:** Comparison of related works.

Ref.	Main Focus	3D Pos.	Attitude	Direction	Advantages	Limitations
[6]	MAND	No	No	Yes	Fast beam alignment with bounded latency	Not FANET-specific; does not model 3D attitude/installation constraints; lacks network-level evaluation
[7]	Cognitive neighbor discovery	No	No	Yes	Reduces expected discovery time via adaptive sector control	Relies on simplified assumptions; mainly focuses on the discovery stage
[8,9]	Reinforcement Learning (RL)-based directional neighbor discovery	No	No	Yes	Low-complexity decentralized schemes can reduce overhead and latency	Training stability/robustness in highly-dynamic scenarios; not tailored to FANETs attitude constraints
[10]	HDND	No	No	Yes	Deterministic spatial rendezvous through continuous scanning	Scanning overhead can be high; not FANET-specific; no 3D attitude modeling
[11]	RL-based discovery without prior neighbor location information	No	No	Yes	Improves discovery efficiency without requiring prior location knowledge	Learning cost and convergence concerns; limited to the discovery phase
[12]	ND-LP/ACI-LP	Partial	No	Yes	Accelerates main-lobe/channel convergence via position prediction	Often based on simplified mobility assumptions; no explicit attitude/installation constraints
[13]	Cross-layer functionality with beam sector directional antenna technique	Partial	No	Yes	Enhances beam tracking and reduces interruption probability	Focuses on beam tracking; limited characterization of large-scale FANETs networking
[14]	MADDRL	No	No	Yes	Interference-aware adaptive transmission and antenna adjustment	Training complexity; limited explainability; not FANET 3D attitude-centric
[15]	Hierarchical beamforming based on hybrid CSI	No	No	Yes	Balances communication performance and interference suppression	Not inherently designed for highly-dynamic FANETs environments
[16]	CPR-TD	Yes	No	No (Omni)	Reduces routing overhead; improves PDR in mission-oriented FANETs	Assumes omnidirectional links; does not address directional connectivity/beam pointing
[17]	BC-DSR	Partial	No	No (Omni)	More robust relay selection under correlated mobility	Omnidirectional assumption; no antenna installation/attitude constraints
This work	Constrained antenna selection and beam pointing control for directional FANETs	Yes	Yes	Yes	Jointly accounts for 3D position and attitude-driven installation constraints	Requires attitude/installation information; introduces additional control/coordination overhead

**Table 2:** Nomenclature and coordinate system definitions.

Symbol	Definition	Description	Note
ψ,θ,γ	Euler Angles	Represent yaw, pitch, and roll, respectively.	The system attitude is defined by the yaw-pitch-roll convention (Y-Z-X sequence). The rotation matrix is calculated as $C_{T}^{B}=M_{1}(\gamma )M_{3}(\theta )M_{2}(\psi )$.
* Seq. *	Rotation Order	Y-Z-X sequence (intrinsic rotations).	
$C_{T}^{B}$	Rotation Matrix	Rotation from the E system to the T system.	
$M_{1},M_{2},M_{3}$	Elementary Matrices	Basic rotation matrices about fundamental axes.	

**Table 3:** Key simulation parameters configuration.

Parameter	Value	Description
Simulation Environment		
Total Simulation Slots	40,000	Total duration logic units
Slot Duration	5 ms	Time interval per slot
Number of Nodes	5, 10, 15, 20, 25, 30	Network scale
Maximum Speed of Node	500 m/s	highly-dynamic property of nodes
Channel Transmission Model	Free Space Path Loss	
Physical Layer (mmWave)		
Carrier Frequency ($f_{c}$)	60 GHz	Operating frequency
System Bandwidth (B)	500 MHz	Channel bandwidth
Transmit Power ($P_{tx}$)	20 dBm	UAV transmission power
Noise Figure (NF)	6 dB	Receiver circuitry noise
Path Loss Exponent (α)	2.1	Propagation environment
Antenna Model		
Main Lobe Gain ($G_{main}$)	25 dBi	High gain for aligned beam
Side Lobe Gain ($G_{side}$)	−5 dBi	Low gain for side lobes
3 dB Beamwidth ($\theta_{3dB}$)	15°	Beam alignment tolerance
Traffic and MAC		
MAC Protocol	TDMA	
Packet Arrival Rate (λ)	0.6	Probability/slot/node (Poisson)
Packet Size	500 kbits	Data packet payload size
Neighbor information update Interval (ms)	5 ✕ *n*	* n * denotes the number of active nodes in the network.

**Table 4:** Physical experiment results.

ID	The Success Rate of Antenna Selection	Beam Pointing Deviation	Packet Delivery Ratio	Average E2E Delay (s)
Test 1	97%	0.012	100%	0.34
Test 2	98.5%	0.02	100%	0.29
Test 3	96%	0.017	100%	0.31

## Data Availability

All data generated during this study are included in this article.
